# Unveiling Hidden Threats: Bacterial Contamination of Frequently Touched Objects and the Biofilm Property of *Staphylococcus aureus* as a Threat to Antibiotic Success

**DOI:** 10.1155/cjid/9929263

**Published:** 2025-12-08

**Authors:** Rakshya Bhandari, Niroj Man Amatya, Prashamsa Bogati, Purnika Sulu, Pratima Baniya

**Affiliations:** ^1^ Department of Medical Laboratory Technology, Nobel College, Pokhara University, Sinamangal, Kathmandu, Nepal, pu.edu.np; ^2^ Department of Medical Microbiology, Nobel College, Pokhara University, Sinamangal, Kathmandu, Nepal, pu.edu.np

**Keywords:** biofilm, CoNS, frequently touched objects, HCAIs, MRSA, MSSA

## Abstract

**Background:**

Frequently touched objects within hospital premises acts as a potential reservoir for healthcare‐associated infections (HCAIs), significantly amplifying the risk when biofilm‐producing bacteria are involved. These bacteria often exhibit multidrug‐resistant (MDR) patterns, complicating the antimicrobial treatments. So, this study intended to determine the bacterial contamination level on frequently touched objects and their antibiotic susceptibility patterns and to determine the relation between biofilm‐producing *Staphylococcus aureus* and multidrug resistance patterns.

**Methods:**

A hospital‐based cross‐sectional study was conducted at different intensive care units (NICU and MICU) and operation theatre of Frontline Hospital, Kathmandu, Nepal. Aseptically, 297 swab samples were collected and cultured, and the isolated bacteria were identified using standard microbiological procedures. Antibiotics susceptibility test done by the Kirby–Bauer disc diffusion method, and biofilm detection was done by the microtiter plate method at 570 nm by using an ELISA reader. For data analysis, SPSS Version 23 was used.

**Results:**

Of total 297 swab samples processed, 31.3% (93/297) showed bacterial growth, yielding a total of 108 isolates. Mixed growth was reported on 16.1% (15/93) samples. The frequency of Gram‐positive and Gram‐negative bacteria was 95.4% (103/108) and 4.6% (5/108), respectively. The main isolates were coagulase‐negative staphylococci (CoNS) 50.9%, followed by *S. aureus* (36.1%), Gram‐positive bacilli (8.3%), *Pseudomonas* species (2.8%), and *Klebsiella* species (1.9%). Out of 39 *S. aureus* isolates, 53.8% (21/39) were MDR and 25 (64.10%) were biofilm producers. Similarly, 35.9% (14/39) were methicillin‐resistant *S. aureus* (MRSA), among which majority 71.4% (10/14) of MRSA recovered as biofilm producers.

**Conclusion:**

A frequently touched object within different intensive care units and operation theatre was found to be contaminated with potential pathogens and normal flora. Bacterial contamination of such objects can contribute to HCAIs and the hands of health professionals can be the mode of cross‐contamination.

## 1. Introduction

Bacterial contamination of frequently touched objects in hospital settings is a critical factor contributing to healthcare‐associated infections (HCAIs) [1]. The most commonly touched objects and surfaces in the patient care zone include bed rails, door‐knobs, trays, tables, nurse call buttons, and drip bottles, which often harbor various bacteria for different durations [2]. These contaminants can be transferred to the hands and equipment of healthcare professionals, thereby transmitting infectious agents to other patients during routine care [3]. The environment of intensive care unit (ICU) and hands of healthcare professionals are linked to potential pathogens causing HCAIs [4–6]. The risk of transmission correlates directly with the duration of bacterial survival on colonized objects. Geographical and environmental factors, such as temperature, humidity, availability of organic matter, ability to form biofilms, and commonly used infection control techniques, influence colonization and survival. Additionally, poor hand hygiene, overcrowding, understaffing, inadequate training of human resources, and insufficient disinfection and fumigation are responsible for this issue [2].

According to the World Health Organization’s Global Report on Infection Prevention and Control, for every hundred patients in acute care hospitals, seven patients in high‐income countries and 15 in low‐ and middle‐income countries will experience at least one HCAI during their hospital stay. On average, one in ten infected individuals will die from HCAIs [7]. In critical care settings, HCAIs are ranked the fifth most common cause of mortality [2]. Globally, more than one million neonatal deaths are recorded annually, with 30%–40% of these deaths among neonates associated with HCAIs in resource‐constrained countries. Each day, HCAIs contribute to antimicrobial resistance, extended hospital stays, long‐term disabilities, significant additional costs for healthcare systems, high expenses for patients and their families, and premature deaths [8].

The organisms responsible for the most HCAIs often originate from the patient’s endogenous flora; however, they can also spread through contact with healthcare personnel, fomites (such as needles and other medical devices), and the surrounding environment (exogenous flora) [9]. Organisms usually found in hospital environments and on surfaces include methicillin‐resistant *Staphylococcus aureus* (MRSA), vancomycin‐resistant Enterococci (VRE)*, Pseudomonas* species, and *Acinetobacter* species [10–12]. Gram‐positive bacteria, including MRSA which is a significant contributor to HCAIs, can persist on dry, nonliving surfaces for several weeks [13, 14]. Recently, there has been increasing evidence of MRSA’s ability to form biofilms, exacerbating the situation in hospitals and contributing to the rise in antibiotic resistance. The ability to form biofilms is a key factor in the virulence of *Staphylococcus* bacteria, leading to persistent and recurring infections associated with medical devices [15]. An increased recalcitrance of biofilm‐associated bacteria often results in frequent treatment failures, making the management of such infections increasingly challenging [16].

The environmental infection control in healthcare facilities is a critical component of preventing HCAIs. Recent recommendations from the Centers for Disease Control and Prevention and the Healthcare Infection Control Practices Advisory Committee include a Category II recommendation to clean and disinfect high‐touch surfaces more frequently than minimal‐touch surfaces [17]. A decade ago, there was a lack of comprehensive quantitative evaluations of how often healthcare personnel come into contact with various room surfaces and the types and levels of bioburden present on these surfaces [18]. However, recent studies indicate an increasing trend in HCAIs due to bioburden on frequently touched objects. Contamination levels can be high, and HCAI pathogens can persist on these surfaces for extended periods, ultimately increasing the bioburden on inanimate objects in hospitals and concomitantly raising infection rates [10, 11, 19]. Additionally, biofilms that tenaciously attach to these surfaces create challenges for removing microbes. A study from Manipal Teaching Hospital revealed that 31.8% of biofilm‐producing *S. aureus* were found on inanimate objects within the hospital [2]. Similarly, another study showed that 22.2% of *S. aureus* were biofilm producers. Such biofilm‐producing bacteria remain viable on inanimate surfaces for extended periods contributing to HCAIs [20]. Overall, our study was designed to assess bacterial contamination on frequently touched objects, along with an investigation of biofilm‐forming MRSA colonization on these objects. Moreover, the antibiotic resistance profiles of the isolated bacteria were assessed. This study aims to inform disinfection strategies in hospital settings to mitigate the risk of HCAIs and provide insights into the judicious use of antibiotics.

## 2. Materials and Methods

A hospital‐based cross‐sectional study was conducted from December 2023 to February 2024 at Frontline Hospital, Kathmandu, which has a capacity of 200 beds and includes various ICUs, such as the Medical ICU (MICU), Neonatal ICU (NICU), and a well‐equipped operation theatre (OT). First week of sample collection was performed on 25^th^ December and it extended up to total ninth week of sample collection on 19^th^ February. Sampling objects were meticulously selected based on their potential for bacterial contamination, specifically those frequently coming into contact with the hands of healthcare professionals, visitors, and patients. Samples were collected from the MICU, NICU, and OT. Sampling was conducted weekly, amidst the rhythm of clinical activities, at approximately 11 am. This choice was not based on cleaning schedule but rather to standardize the sampling time without interference in clinical activities and to ensure the comparability of results from midmorning (11 am) was selected.

### 2.1. Specimen Collection

A total of 297 environmental samples were collected from frequently touched inanimate objects such as bed rails (*n* = 54), drip bottles (*n* = 10), stethoscope (*n* = 27), sphygmomanometer (*n* = 09), trays (*n* = 18), table (*n* = 27), light switches (*n* = 27), remote control (*n* = 27), stretcher (*n* = 09), sink tap (*n* = 18), door knobs (*n* = 27), cradle (*n* = 09), weighing machine (*n* = 09), anesthesia machine (*n* = 09), hot air blower (*n* = 09), and vacuum suction devices (*n* = 08). Samples were collected by rubbing a sterile swab premoistened with peptone water across a surface area of 2 cm^2^. After sampling, it was immediately transported to the laboratory for further processing. During the sampling process, each object was clearly labeled and utmost precautions were taken to prevent contamination from the collector’s hands.

### 2.2. Bacterial Isolation and Identification

Swab samples were aseptically streaked onto MacConkey agar and Blood agar. Culture plates were incubated at 37°C for 24–48 h, and the bacterial isolates were identified through standard microbiological procedures such as colony morphology, Gram staining, and biochemical reactions [21].

### 2.3. Antibiotic Susceptibility Test

An antibiotic susceptibility test was performed by the Kirby–Bauer disc diffusion method on Muller–Hinton agar plates based on CLSI guidelines. A test organism was made equivalent to 0.5 McFarland standard, and a lawn culture was performed on MHA plate [13].

The antibiotics tested were amikacin (30 µg), cefepime (30 µg), cefotaxime (30 µg), cefoxitin (30 µg), cefpodoxime (10 µg), ceftriaxone (30 µg), chloramphenicol (30 µg), ciprofloxacin (5 µg), cotrimoxazole (25 µg), erythromycin (15 µg), gentamicin (10 µg), imipenem (10 µg), meropenem (10 µg), penicillin G (10 µg), piperacillin (100 µg), piperacillin/tazobactam (100/10 µg), and tigeycycline (15 µg). Bacterial isolates which are resistant to minimum one agent in three or more than three antibiotic groups were categorized as multidrug resistant (MDR) [22]. MRSA isolates were detected by the cefoxitin (30 µg) disc diffusion method [13]. The diameter of zone of inhibition was measured with a ruler, and results were reported as susceptible (S), intermediate (I), or resistant (R) based on CLSI guidelines. *S. aureus* ATCC 25923 was used as a quality control strain.

### 2.4. Biofilm Formation Assay

Biofilm‐forming property of *S. aureus* isolates were detected by the standard microtiter plate method [23]. *S. aureus* was grown in 96‐well microtiter plate containing trypticase soy broth with 2% glucose at 37°C for 48 h, and the optical density was determined with the help of ELISA reader at 570 nm after staining with crystal violet. The results were interpreted as biofilm producers and biofilm nonproducers based on optical density values [24].

### 2.5. Statistical Analysis

Data entry and analysis were done by using a Statistical Package for the Social Sciences (SPSS), Version 23. A Chi‐square test was applied on categorical data, with a *p* value less than 0.05 considered as significant.

## 3. Result

A total of 297 swab samples were collected for our study from the MICU, NICU, and OT. Among these samples, 93 (31.3%) samples exhibited bacterial growth, with polymicrobial growth noted in 15 (16.1%) samples, resulting in a total of 108 isolates. The remaining 204 (68.7%) samples did not show any growth. The number of samples collected from each department is presented in Supporting Table 1. A majority of the sampling objects showed a predominance of Gram‐positive bacteria, comprising 95.4% (103/108) of the isolates, compared with 4.6% (5/108) for Gram‐negative bacteria. Most of the bacteria were reported from the MICU (53.7%), followed by the NICU (35.2%) and OT (11.1%). The number of Gram‐positive and Gram‐negative isolates from each department, with respect to sampling objects, is shown in Supporting Table 2. The most common isolates were coagulase‐negative staphylococci (CoNS), followed by *S. aureus*, Gram‐positive bacilli, *Pseudomonas* spp., and *Klebsiella* spp. (Table 1). The majority of S. *aureus* and CoNS were recovered from bed rails, while *Pseudomonas* spp. were found to be colonizing sink taps, and *Klebsiella* spp. were isolated from both sink taps and bed rails.

**Table 1:** Frequency of etiological agents.

S.N.	Organisms	Total isolates (*n* = 108)			Total
		MICU (%)	NICU (%)	OT (%)	
1	*Staphylococcus aureus*	24 (22.2%)	8 (7.4%)	7 (6.5%)	39 (36.1%)
2	*Klebsiella* spp.	1 (0.9%)	1 (0.9%)	0	2 (1.9%)
3	*Pseudomonas* spp.	1 (0.9%)	2 (1.9%)	0	3 (2.8%)
4	CoNS	26 (24.0%)	24 (22.22%)	5 (4.63%)	55 (50.93%)
5	Gram‐positive bacilli	6 (5.55%)	3 (2.78%)	0	9 (8.33%)
	Total	58 (53.7%)	38 (35.2%)	12 (11.1%)	108 (100%)

Antibiotic susceptibility tests were done for bacteria having clinical relevance. Among *S. aureus*, the percentage of MRSA and methicillin‐sensitive *S. aureus* (MSSA) was 35.9% (14/39) and 64.1% (25/39) respectively. The antibiotic resistance patterns of *S. aureus* are depicted in Table 2. For *Pseudomonas* spp., ciprofloxacin and imipenem were susceptible, while the resistant rate for amikacin, cefepime, ceftriaxone, meropenem, piperacillin, and piperacillin_tazobactam was 33.3%. In case of *Klebsiella* spp., except for cefotaxime and cepodoxime, rest other antibiotics successfully were effective in killing the bacterium.

**Table 2:** Antibiotic resistance pattern of *S. aureus* (MRSA and MSSA) and CoNS isolates.

Antibiotics	*S. aureus* (*n* = 39) Frequency (%)	MRSA (*n* = 14) Frequency (%)	MSSA (*n* = 25) Frequency (%)	CoNS (*n* = 55) Frequency (%)
Amikacin	0	0	0	3 (4.45)
Cefoxitin	14 (35.9)	—	0	18 (32.72)
Cefotaxime	15 (38.5)	—	1 (4.0)	17 (30.91)
Ceftriaxone	15 (38.5)	—	1 (4.0)	18 (32.72)
Cefpodoxime	29 (74.4)	—	15 (60.0)	37 (67.27)
Chloramphenicol	0	0	0	0
Ciprofloxacin	3 (7.7)	3 (21.4)	0	9 (16.36)
Cotrimoxazole	5 (12.8)	2 (14.3)	3 (12.0)	9 (16.36)
Erythromycin	27 (69.2)	13 (92.9)	14 (56.0)	45 (81.82)
Penicillin G	35 (89.7)	—	21 (84.0)	50 (90.91)
Tigeycycline	1 (2.6)	1 (7.1)	0	0

**Figure 1:**
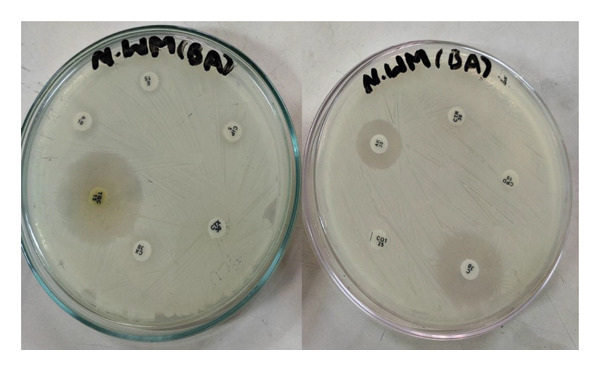
Antibiotic susceptibility pattern of MRSA.

Altogether 53 MDR bacteria were isolated (23 from MICU, 24 from NICU, and 6 from OT). Our study indicated that the percentage of MDR in CoNS, *S. aureus*, and *Pseudomonas* spp. were 56.4% (31/55), 53.8% (21/39), and 33.3% (1/3), respectively, while no MDR was reported from *Klebsiella* spp. The overall MDR status of organisms is presented in Table 3, and Figure 1 shows the antibiotic susceptibility pattern of MDR *S. aureus*. Among 39 isolated *S. aureus*, 25 (64.1%) were biofilm producers and remaining 14 (35.9%) were biofilm nonproducers. The majority of MRSA (71.4%; 10/14) and MSSA (60%; 15/25) were biofilm producers, which are presented in a Table 4. The Chi‐square test showed that the correlation between MDR status due to MRSA and biofilm producers was statistically insignificant (*p* = 0.475).

**Table 3:** MDR status of different organisms.

	CoNS	*S. aureus*	*Pseudomonas* spp.	*Klebsiella* spp.	Total
MDR	31	21	1	—	53
Non‐MDR	24	18	2	2	46
Total	55	39	3	2	99

Abbreviations: MDR, multidrug resistant; non‐MDR, nonmultidrug resistant.

**Table 4:** Biofilm formation in MRSA and MSSA isolates.

Organisms	Biofilm production		Total
	Positive	Negative	
MRSA	10 (71.4%)	04 (28.6%)	14
MSSA	15 (60%)	10 (40%)	25
Total	25 (64.1%)	14 (35.9%)	39

## 4. Discussion

HCAIs are among the most prevalent adverse events occurring during the delivery of care [8]. Several factors may contribute to the prevalence of HCAIs, with bacterial contamination of frequently touched objects in hospital environments being a critical factors contributing to HCAIs [1].

The results of our study at Frontline Hospital, Nepal, showed that the overall bacterial contamination rate is 36.4% (108 out of 297 samples), in which the number of Gram‐positive isolates (103/108; 95.4%) were more than Gram‐negative isolates (5/108; 4.6%). These findings align with a similar study conducted in Northwest Ethiopia, which reported an overall bacterial growth rate of 39.6% and dominancy of Gram‐positive isolates (81.6%) were higher than Gram‐negative isolates (18.4%) [25]. In contrast, a study conducted in Iran showed slightly deviated results, showing a lower contamination rate of Gram‐positive (60.7%) and a higher contamination rate of Gram‐negative (39.3%) isolates than ours [26]. But research from India indicated a reversed rate, with higher rate of infection in Gram‐negative compared with Gram‐positive isolates [27]. The differences in these findings may be attributed to several factors such as temperature, humidity, nutrient availability, and specific location of sampling sites/objects. The dominance of Gram‐positive bacteria can be explained by their innate ability to survive in abiotic hospital settings for several days to months due to their lack of a lipid‐dominant, desiccation‐prone outer membrane [28].

The highest bacterial contamination was observed in frequently touched objects in MICU (53.7%), followed by NICU (35.24%) and OT (11.1%). In contrast, studies conducted at Manipal Teaching Hospital and Nigerian Hospital showed higher bacterial contamination in the NICU accounting 74.6% [29] and 67.6% [30], respectively. Notably, no bacterial contamination was found in a study conducted in ICU of Pakistan [31]. The differences in geographical location, hospital environment, and patient load, etc. can be the factors for contrast in these study results.

The highest contamination recorded in MICU may be attributed to several factors, as critically ill patients necessitate frequent care from healthcare professionals. The hands of healthcare professionals are the primary source of transmission of HCAIs [26]. Additional contributing factors may include invasive procedures such as insertion of catheters, ventilators, or central lines, all of which pose an infection risk. Moreover, frequent interactions with visitors and prolonged hospital stays expose patients to the hospital environment and potential pathogens that may facilitate the transmission of HCAIs. In our study, the common potential pathogens isolated were CoNS, *S. aureus, Pseudomonas* spp., and *Klebsiella* spp. Similar findings have been reported in Nigerian Hospital [30] and in Northwest Ethiopia [25]. Majority of CoNS were reported from bed rails, door knobs, and light switches. Similarly, majority of *S. aureus* were isolated from bedrails, remote switches, stretchers, and trays, while the least number was obtained from drip bottles.

Regarding the antimicrobial resistance profile of CoNS isolates, our results showed high drug resistance to some of tested antibiotics such as penicillin G and erythromycin, followed by cefpodoxime and ceftriaxone. Conversely, tigeycycline, chloramphenicol, cotrimoxazole, and amikacin were found to be the most susceptible antibiotics, which is in line with similar resistance rates reported from other study conducted in Ethiopia [28]. For *S. aureus*, penicillin G and erythromycin were the least effective antibiotics, while no resistance was observed for amikacin and chloramphenicol, which align with study conducted in Ethiopia [28]. In case of MRSA, higher resistance was reported for erythromycin and ciprofloxacin, while amikacin and chloramphenicol exhibited no resistance.

Among the isolated *S. aureus*, 53.8% (21/39) were confirmed MDR and remaining 46.1% (18/39) were non‐MDR. Similarly, 35.9% (14/39) were MRSA and rest 64.1% (25/39) were found to be MSSA. A similar finding was reported in a study conducted in NICU, which identified 33.3% MRSA [29], whereas another study by Bhatta et al. reported higher percentage of 54.4% MRSA, with 45.5% reported as MSSA [20].

Infection with MDR bacteria can significantly increase the likelihood of antibiotic failure and ineffective treatment. *S. aureus* can persist for several days in hospital settings due to its ability to form biofilms on inanimate objects [2]. In our study, among 39 isolated *S. aureus*, 64.1% (25/39) were confirmed as biofilm producers, of which 10 were MRSA, i.e., the majority of MRSA isolates (71.4% (10/14)) were biofilm producers. A similar study by Bhatta et al. reported that 62.5% of MRSA were biofilm producer, which is slightly lower than our findings [2]. Similarly, another study from Manipal Teaching Hospital reported that majority of MRSA were biofilm producing (66.7%), which is comparable to our results [29]. Another study by Belbase et al. showed a higher rate of MRSA among biofilm producers compared with biofilm nonproducer strains [32]. The antibiotic resistance in bacterial strains within biofilms can increase by as much as 1000 times. This heightened resistance is likely attributed to multiple factors such as difficulty of antibiotic penetration through biofilms, slow growth rate of bacteria, and presence of mechanisms that degrade antibiotics [33]. Biofilms are complex and adaptive, so a multifaceted approach is necessary for its effective management.

Our study findings reported 4.6% Gram‐negative isolates, which includes species of *Pseudomonas* (2.8%) and *Klebsiella* (1.9%). A similar result was reported in a pediatric ward of Nigerian Hospital, where *Pseudomonas* spp. were found to be 1.3% [30] and Bara reported *Klebsiella* spp. to be 4.3% [1]. Among five Gram‐negative isolates, 20% (1/5) were confirmed to be MDR. Antibiotics ciprofloxacin and imipenem were susceptible to *Pseudomonas* spp. while the resistance rate for amikacin, cefepime, ceftriaxone, meropenem, piperacillin, and piperacillin_tazobactam was 33.3%. For *Klebsiella* spp., except for cefotaxime and cepodoxime, rest other antibiotics successfully kill or inhibit the bacterium. Similar findings were reported by Bara [1] and Bhatta et al. [29].

The clinical practices in ICUs includes the mechanical and surgical management of critically ill patients, along with excessive use of antibiotics, making them more vulnerable to an increased risk of HCAIs and the growth of MDR bacteria in hospital environment. Such a scenario has the unintended consequences of making hospital infection control and patient care even more difficult [1], especially worsened in developing countries like Nepal, without access to other alternative chemotherapeutic options for management and treatment of infections related to MDR. Apparently, hospital environments may appear clean but they can still harbor bacteria, making complete sterility difficult to achieve. Both healthcare professionals and patients can be potential source of pathogens and they facilitate cross‐transmission. However, strengthening environmental cleaning and routine monitoring of highly touched objects may further enhance infection prevention strategic in healthcare settings. Also, by implementing touch‐free technologies such as sensor‐operated faucets for hand washing, automatic sensor doors and lights, and touch‐free elevator controls can help prevent the bacterial contamination to some extent in healthcare settings.

The purpose of this study is to ascertain the significance of bacterial colonization on frequently touched objects in hospital environment and its contribution to HCAIs. Our study findings shed insight into different pathogenic bacteria present on frequently touched objects in hospitals and their antibiotic resistance profiles with biofilm‐forming property of *S. aureus.* This information may help predict the present landscape of HCAIs and the rising peril of antibiotic failure.

## 5. Conclusion

The highest level of bacterial contamination was found on frequently touched objects in MICU followed by NICU and OT. Isolation of potential pathogens, such as CoNS, *S. aureus*, *Pseudomonas* spp., and *Klebsiella* spp., from these objects provides essential baseline information regarding the degree of contamination by environmental isolates, which could be a potential cause of HACIs.

The presence of biofilm‐producing MRSA from the sites is a potential threat to antibiotic treatment. The present study emphasizes the need for modification of cleaning/disinfection procedures and judicious use of antibiotics in order to reduce the risk of HCAIs and antibiotic failure. Provision of no‐touch techniques for door and taps/faucets and periodic microbiological surveillance in critical care units and operation theatre could possibly reduce the transmission of potential pathogens.

## Ethics Statement

An ethical approval was taken from Nobel College Institutional Review Committee (Ref. no. 080/081/283), and written consent was obtained from Frontline Hospital, Kathmandu, Nepal.

## Disclosure

All the authors have read and accepted the article for publication.

## Conflicts of Interest

The authors declare no conflicts of interest.

## Author Contributions

Rakshya Bhandari contributed to sample collection, laboratory processing, data entry, data analysis, and drafting of manuscript. Niroj Man Amatya contributed to study design, streamlining lab processing, assisted in data analysis, and refined the article. Prashamsa Bogati, Purnika Sulu, and Pratima Baniya contributed to sample collection and processing.

## Funding

No funding was received for this manuscript.

## Supporting Information

Supporting Table 1: Unit‐wise distribution of samples.

Supporting Table 2: Bacterial isolates from each department.

## Supporting information


**Supporting Information** Additional supporting information can be found online in the Supporting Information section.

## Data Availability

The data that support the findings of this study are available from the corresponding author upon reasonable request.
