# Supplementary material for: Unveiling Hidden Threats: Bacterial Contamination of Frequently Touched Objects and the Biofilm Property of Staphylococcus aureus as a Threat to Antibiotic Success
Source: Can J Infect Dis Med Microbiol. 2025 Dec 8;2025:9929263. doi: 10.1155/cjid/9929263 (PMC12747103; doi:10.1155/cjid/9929263)
Supplement: Supplementary file 1 — Supporting Information Additional supporting information can be found online in the Supporting Information section. [file CJID-2025-9929263-s001.docx]

Supplementary table 1.

Supplementary table 1: Unit wise distribution of samples

| **S.N.** | **Sampling Objects** | **Number of sample from** | | |
| --- | --- | --- | --- | --- |
|  |  | **MICU** | **NICU** | **OT** |
| 1 | First bedrail | 9 | 9 | 9 |
| 2 | Second bedrail | 9 | 9 | 9 |
| 3 | First drip bottle | 5 | - | - |
| 4 | Second drip bottle | 5 | - | - |
| 5 | Stethoscope | 9 | 9 | 9 |
| 6 | Sphygmomanometer | 9 | - | - |
| 7 | Trays | 9 | 9 | - |
| 8 | Table | 9 | 9 | 9 |
| 9 | Light switch | 9 | 9 | 9 |
| 10 | Remote control | 9 | 9 | 9 |
| 11 | Stretcher | 9 | - | - |
| 12 | Sink tap | 9 | 9 | - |
| 13 | Door knobs | 9 | 9 | 9 |
| 14 | Cradle | - | 9 | - |
| 15 | Weighing machine | - | 9 | - |
| 16 | Anesthesia machine | - | - | 9 |
| 17 | Hot air blower | - | - | 9 |
| 18 | First vacuum suction | 4 | - | - |
| 19 | Second vacuum suction | 4 | - | - |
|  | Total | 117 | 99 | 81 |

Supplementary table 2.

Supplementary table 2 :Bacterial isolates from each department

| **S.N.** | **Sampling Objects** | **Bacteria isolated from** | | | | | |
| --- | --- | --- | --- | --- | --- | --- | --- |
|  |  | **MICU** | | **NICU** | | **OT** | |
|  |  | **GP** | **GN** | **GP** | **GN** | **GP** | **GN** |
| 1 | First bedrail | 3 | - | 3 | - | 3 | - |
| 2 | Second bedrail | 2 | 1 | 4 | - | 1 | - |
| 3 | Door knobs | 7 | - | 3 | - | - | - |
| 4 | Table | 1 | - | 2 | - | 1 | - |
| 5 | Stethoscope | 3 | - | 1 | - | 1 | - |
| 6 | Light | 8 | - | 5 | - | - | - |
| 7 | Remote | 7 | - | 1 | - | 1 | - |
| 8 | Sink tap | 5 | 1 | 4 | 3 | - | - |
| 9 | Tray | 2 | - | 4 | - | - | - |
| 10 | First drip bottle | - | - | - | - | - | - |
| 11 | Second drip bottle | 2 | - | - | - | - | - |
| 12 | Sphygmomanometer | 2 | - | - | - | - | - |
| 13 | Stretcher | 9 | - | - | - | - | - |
| 14 | Cradle | - | - | 3 | - | - | - |
| 15 | Weighing machine | - | - | 5 | - | - | - |
| 16 | Anesthesia machine | - | - | - | - | 1 | - |
| 17 | Hot air blower | - | - | - | - | 4 | - |
| 18 | First Vacuum suction | 2 | - | - | - | - | - |
| 19 | Second vacuum suction | 3 | - | - | - | - | - |
|  | Total | 56 | 2 | 35 | 3 | 12 | - |
|  |  | 49.57% | | 38.38% | | 14.48% | |

GP: Gram-positive; GN: Gram-negative
